# PPARα activation promotes liver progenitor cell-mediated liver regeneration by suppressing YAP signaling in zebrafish

**DOI:** 10.1038/s41598-023-44935-5

**Published:** 2023-10-25

**Authors:** Minwook Kim, Juhoon So, Donghun Shin

**Affiliations:** Department of Developmental Biology, McGowan Institute for Regenerative Medicine, Pittsburgh Liver Research Center, University of Pittsburgh, 3501 5th Ave. #5063, Pittsburgh, PA 15260 USA

**Keywords:** Hepatology, Differentiation

## Abstract

Despite the robust regenerative capacity of the liver, prolonged and severe liver damage impairs liver regeneration, leading to liver failure. Since the liver co-opts the differentiation of liver progenitor cells (LPCs) into hepatocytes to restore functional hepatocytes, augmenting LPC-mediated liver regeneration may be beneficial to patients with chronic liver diseases. However, the molecular mechanisms underlying LPC-to-hepatocyte differentiation have remained largely unknown. Using the zebrafish model of LPC-mediated liver regeneration, *Tg*(*fabp10a:pt-β-catenin*), we present that peroxisome proliferator-activated receptor-alpha (PPARα) activation augments LPC-to-hepatocyte differentiation. We found that treating *Tg*(*fabp10a:pt-β-catenin*) larvae with GW7647, a potent PPARα agonist, enhanced the expression of hepatocyte markers and simultaneously reduced the expression of biliary epithelial cell (BEC)/LPC markers in the regenerating livers, indicating enhanced LPC-to-hepatocyte differentiation. Mechanistically, PPARα activation augments the differentiation by suppressing YAP signaling. The differentiation phenotypes resulting from GW7647 treatment were rescued by expressing a constitutively active form of Yap1. Moreover, we found that suppression of YAP signaling was sufficient to promote LPC-to-hepatocyte differentiation. Treating *Tg*(*fabp10a:pt-β-catenin*) larvae with the TEAD inhibitor K-975, which suppresses YAP signaling, phenocopied the effect of GW7647 on LPC differentiation. Altogether, our findings provide insights into augmenting LPC-mediated liver regeneration as a regenerative therapy for chronic liver diseases.

## Introduction

As a highly regenerative organ, the liver regenerates in two distinct ways: (1) hepatocyte proliferation and (2) cell fate conversion^1^. Upon two-thirds partial hepatectomy (PHx) in rodents, the lost liver mass recovers within 8 days through hepatocyte proliferation^2^. However, despite this robust regenerative capacity, prolonged liver damage can impair the ability of hepatocytes to proliferate. In such situations, the liver co-opts cell fate conversion-mediated regeneration, thereby generating hepatocytes from biliary epithelial cells (BECs) or through liver progenitor cells (LPCs)^1^. When hepatocyte proliferation is severely impaired in rodents pharmacologically^3, 4^, genetically^5–8^, or by long-term chronic injuries^9, 10^, BECs convert to hepatocytes, thereby contributing to liver regeneration. This phenomenon also occurs in zebrafish when nearly all hepatocytes are ablated^11–13^ or when oncogenes are overexpressed in hepatocytes^14^. With these rodent and zebrafish models, some of the molecular mechanisms underlying the cell fate conversion-mediated liver regeneration have been identified^15–17^, but the mechanisms are still largely unknown.

The correlation between the number of LPCs and the severity of liver diseases in patients^18^ suggests that LPCs fail to differentiate efficiently into hepatocytes in the diseased liver. However, if the differentiation can be restored, it may promote cell fate conversion-mediated liver regeneration, contributing to liver recovery in patients. Thus, it is clinically important to understand the molecular mechanisms of LPC-to-hepatocyte differentiation and to identify means to promote the differentiation. Given this clinical significance, we sought to identify compounds that can promote LPC-to-hepatocyte differentiation and, herein, discovered the peroxisome proliferator-activated receptor alpha (PPARα) agonist GW7647 as such a compound.

Although PPARα is well known to control lipid metabolism by regulating the expression of multiple genes important for the metabolism^19, 20^, PPARα also controls various biological processes by interacting with multiple signaling pathways. For example, following 2/3 PHx, PPARα facilitates the nuclear translocation of YAP in hepatocytes, thereby enhancing YAP signaling in the hepatocytes and promoting hepatocyte proliferation^21^. Moreover, PPARα promotes cardiomyocyte maturation by enhancing Yap1 expression^22^. In addition to this crosstalk with YAP signaling, PPARα interacts with Notch and WNT signaling. PPARα controls vasculoprotection and vascular remodeling by enhancing Notch signaling through fatty acid oxidation (FAO) in endothelial cells^23^. PPARα also protects diabetic kidneys from renal fibrosis by blocking WNT signaling in renal proximal tubules^24^.

We recently reported that farnesoid X receptor (FXR) suppresses LPC-to-hepatocyte differentiation in zebrafish^15^. Given that FXR and PPARα play opposite roles in multiple processes in the liver, including FAO, gluconeogenesis, and autophagy^20^, we hypothesized that PPARα activation could promote LPC-to-hepatocyte differentiation. By testing this hypothesis, we here present that PPARα activation indeed promotes LPC-to-hepatocyte differentiation by suppressing YAP signaling.

## Results

### PPARα activation promotes LPC-mediated liver regeneration, particularly LPC-to-hepatocyte differentiation

**Figure 1:**
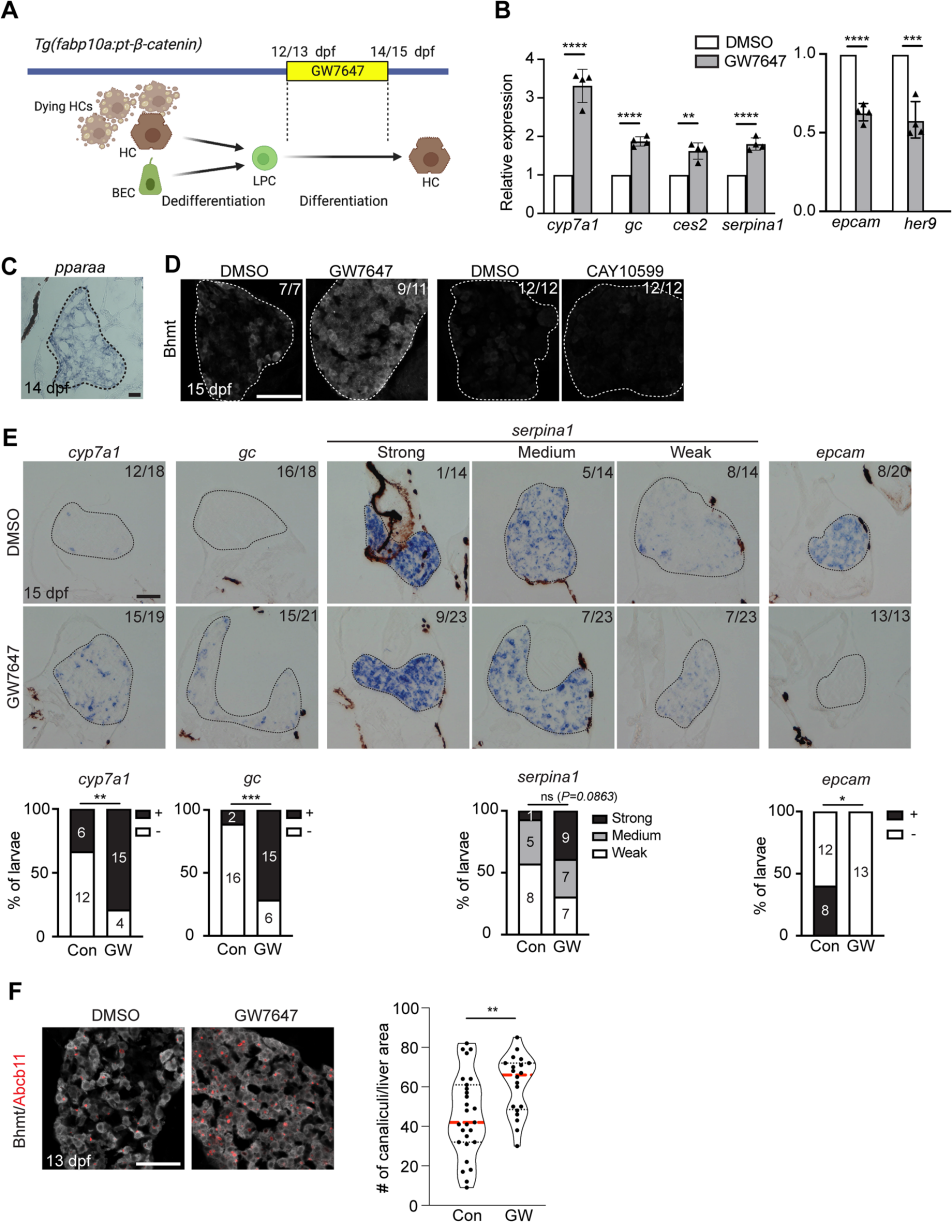
PPARα activation promotes LPC-mediated liver regeneration. (**A**) Scheme illustrating LPC-mediated liver regeneration in *Tg*(*fabp10a:pt-β-catenin*) fish and GW7647 treatment stage. (**B**) qRT-PCR data showing the relative expression levels of hepatocyte (*cyp7a1*, *gc*, *serpina1*, *ces2*) and BEC (*her9*, *epcam*) markers between DMSO- and GW7647-treated *Tg*(*fabp10a:pt-β-catenin*) livers at 14 dpf. (**C**) Section in situ hybridization images showing *pparaa* expression in *Tg*(*fabp10a:pt-β-catenin*) livers at 14 dpf. (**D**) Immunofluorescence images showing Bhmt expression in 15-dpf *Tg*(*fabp10a:pt-β-catenin*) livers treated with 1 μM GW7647 or 7 μM CAY10599. (**E**) Section in situ hybridization images showing the expression of hepatocyte (*cyp7a1*, *gc*, *serpina1*) and BEC (*epcam*) markers in 15-dpf *Tg*(*fabp10a:pt-β-catenin*) livers. For quantification, larvae were grouped into +/− (*cyp7a1*, *gc*, *epcam*) or strong/medium/weak (*serpina1*) based on gene expression levels. (**F**) Immunofluorescence images showing the expression of Abcb11 (red) and Bhmt (grey) in 13-dpf *Tg*(*fabp10a:pt-β-catenin*) livers. Violin plot graphs show the quantification of the number of Abcb11^+^ canaliculi per liver area; median and quartiles are indicated by red dashed and black dotted lines, respectively. Dashed lines outline the livers (**C**–**E**). Numbers in the upper right corner indicate the proportion of larvae exhibiting the phenotype shown (**D**, **E**). Data are represented as mean ± SD (**B**). *P < 0.05, **P < 0.01, ***P < 0.001, ****P < 0.0001; statistical significance was calculated using an unpaired two-tailed *t*-test (**B**, **F**), Fisher’s exact test (**E**: *cyp7a1, gc, epcam*), and chi-square test (**E**: *serpina1*). Scale bars, 50 μm.

To investigate the effect of PPARα activation on LPC-mediated liver regeneration, we used the *Tg*(*fabp10a:pt-β-catenin*)^*s704*^ line, which expresses an oncogenic, stable form of Xenopus β-catenin in hepatocytes under the hepatocyte-specific *fabp10a* promoter^14^. The hepatocyte-specific expression of the oncogene induces senescence and apoptosis in hepatocytes, which elicit LPC activation and inflammation in the liver. BECs and surviving hepatocytes dedifferentiate into LPCs, and the LPCs gradually and slowly differentiate into hepatocytes^14^. Since LPCs are predominant in the *Tg*(*fabp10a:pt-β-catenin*) liver between 12/13 dpf and 14/15 dpf, we treated *Tg*(*fabp10a:pt-β-catenin*) larvae with 1 μM GW7647, a potent PPARα agonist^25^, for 2 days from 12 or 13 dpf (Fig. 1A). As expected, PPARα target genes were upregulated in GW7647-treated *Tg*(*fabp10a:pt-β-catenin*) livers compared with DMSO-treated *Tg*(*fabp10a:pt-β-catenin*) livers (Fig. S1A). Notably, the expression of hepatocyte marker genes (*cyp7a1, gc, ces2, serpina1*) was enhanced, and the expression of BEC marker genes (*epcam* and *her9*) was reduced in GW7647-treated *Tg*(*fabp10a:pt-β-catenin*) livers, suggesting enhanced LPC-to-hepatocyte differentiation (Fig. 1B). In wild-type livers, GW7647 treatment did not affect the expression of the hepatocyte marker genes (data not shown). In the zebrafish genome, there are two PPARα genes, *pparaa* and *pparab*. *pparaa*, but not *pparab*, expression was detected in 14-dpf *Tg*(*fabp10a:pt-β-catenin*) livers by in situ hybridization (Fig. 1C, Fig. S1B; data not shown). Given the possibility that GW7647 may activate PPARγ as well as PPARα^26^, we treated *Tg*(*fabp10a:pt-β-catenin*) larvae with CAY10599, a potent PPARγ agonist. Unlike GW7647 treatment, CAY10599 treatment did not enhance the expression of Bhmt, another hepatocyte marker^27^, in *Tg*(*fabp10a:pt-β-catenin*) livers (Fig. 1D). Altogether, these data suggest that GW7647 treatment in *Tg*(*fabp10a:pt-β-catenin*) larvae promotes LPC-to-hepatocyte differentiation through Pparaa activation.

We next further explored the effect of PPARα activation on LPC-to-hepatocyte differentiation. Section in situ hybridization analysis revealed the enhanced expression of hepatocyte marker genes (*cyp7a1, gc, serpina1*) and the reduced expression of the BEC marker *epcam* in GW7647-treated *Tg*(*fabp10a:pt-β-catenin*) livers (Fig. 1E), consistent with the qRT-PCR data. Moreover, we examined the formation of bile canaliculi by assessing the expression of Abcb11b, a bile salt export pump in the bile canaliculi of hepatocytes^28^. The number of Abcb11b^+^ canaliculi per liver area was significantly increased in GW7647-treated *Tg*(*fabp10a:pt-β-catenin*) livers (Fig. 1F), suggesting promoted LPC-to-hepatocyte differentiation, with the caveat that the increased number could be due to reduced hepatocyte size. Altogether, these data indicate that PPARα activation promotes LPC-to-hepatocyte differentiation.

### PPARα activation suppresses YAP signaling in *Tg*(*fabp10a:pt-β-catenin*) livers

**Figure 2:**
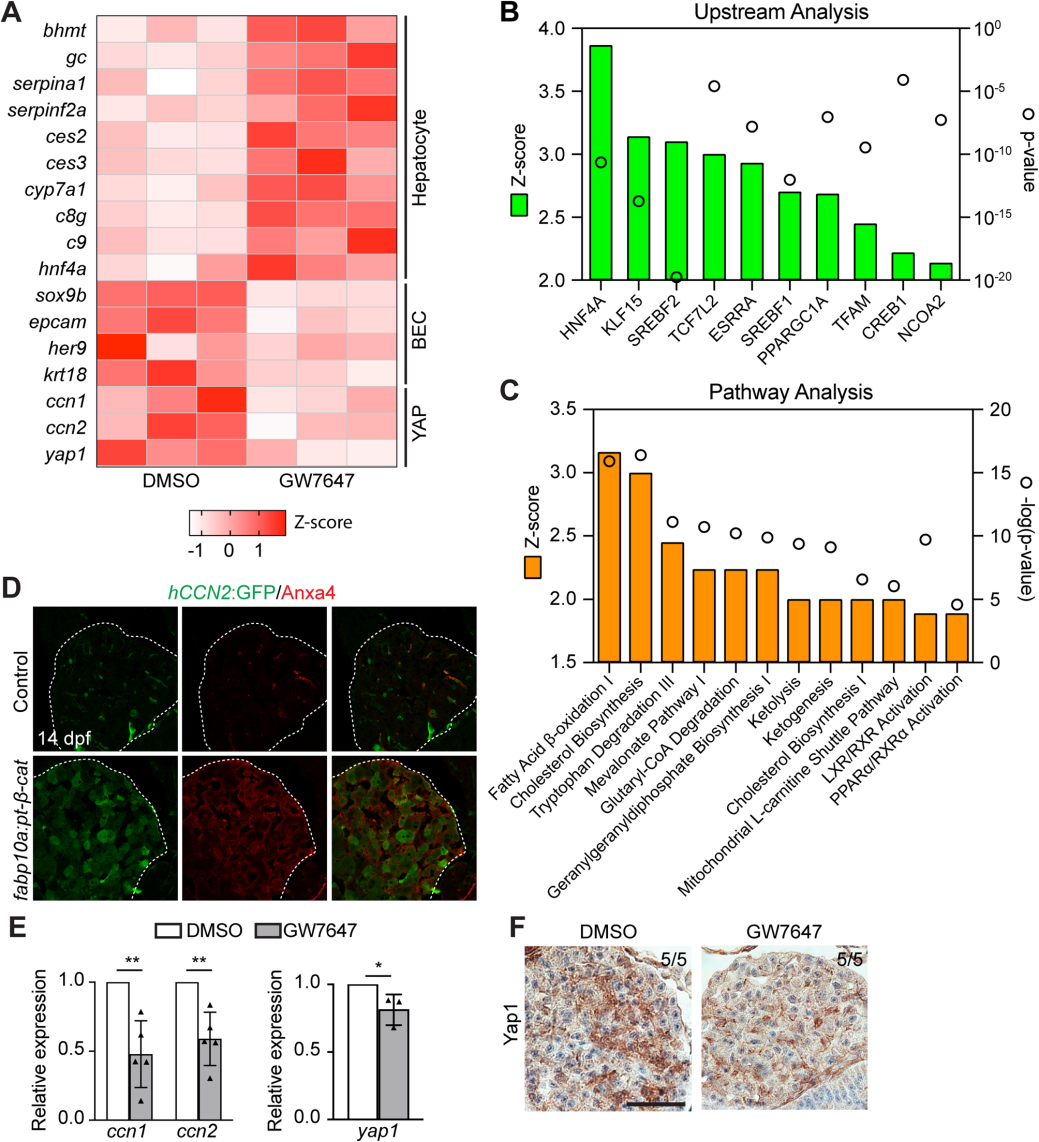
PPARα activation reduces YAP signaling in *Tg*(*fabp10a:pt-β-catenin*) livers. (**A**) Heatmap showing the differentially expressed hepatocyte, BEC, and YAP-related genes between DMSO- and GW7647-treated groups. Each column represents an independent sample (n = 3). (**B**) Upstream analysis showing the top 10 significantly upregulated gene sets of transcriptional regulators in the GW7647-treated group. (**C**) Pathway analysis showing biological pathways significantly induced in the GW7647-treated group. (**D**) Immunofluorescence images showing the expression of *hCCN2*:GFP and Anxa4 in control and *Tg*(*fabp10a:pt-β-catenin*) livers at 14 dpf. Dashed lines outline the livers. (**E**) qRT-PCR data showing the relative expression levels of two YAP target genes (*ccn1*, *ccn2*) and *yap1* between DMSO- and GW7647-treated *Tg*(*fabp10a:pt-β-catenin*) livers at 14 dpf. **P < 0.01; statistical significance was calculated using an unpaired two-tailed *t*-test. (**F**) Immunohistochemistry data showing Yap1 expression in 14-dpf *Tg*(*fabp10a:pt-β-catenin*) livers treated with GW7647. Scale bars, 50 μm.

We compared gene expression profiles between DMSO- and GW7647-treated *Tg*(*fabp10a:pt-β-catenin*) livers by performing RNA-sequencing. Consistent with the above data (Fig. 1), hepatocyte and BEC marker genes were upregulated and downregulated, respectively, in GW7647-treated *Tg*(*fabp10a:pt-β-catenin*) livers compared with DMSO-treated *Tg*(*fabp10a:pt-β-catenin*) livers (Fig. 2A). Moreover, the upstream transcriptional regulator analysis with the Ingenuity Pathway Analysis revealed that gene sets regulated by the transcriptional factors that control hepatocyte differentiation, such as HNF4A^29^ and KLF15^30^, were significantly upregulated in GW7647-treated *Tg*(*fabp10a:pt-β-catenin*) livers (Fig. 2B), supporting that PPARα activation promotes LPC-to-hepatocyte differentiation.

The pathway analysis also revealed that lipid metabolism-related pathways were enhanced in GW7647-treated *Tg*(*fabp10a:pt-β-catenin*) livers (Fig. 2C), as confirmed by qRT-PCR analysis (Fig. S1A). Given that FAO is one of the main target pathways of PPARα^20^ (Fig. 2C) and that FAO regulates cell differentiation in injured kidneys^31^ and vessels^23^, we tested if PPARα activation promotes LPC-to-hepatocyte differentiation by enhancing FAO. To suppress FAO, we used Etomoxir that inhibits Cpt1a^32^, the rate-limiting enzyme in FAO. However, Etomoxir treatment did not diminish the effect of PPARα activation on LPC-to-hepatocyte differentiation in *Tg*(*fabp10a:pt-β-catenin*) larvae (Fig. S1C). In addition, *ppargc1a*, a co-activator of PPARα in the induction of FAO-related genes^33^, mutant fish exhibited the same effect of GW7647 on LPC differentiation as their wild-type siblings (Fig. S1D). To further explore candidate pathways that mediate the effect of PPARα activation, we examined the expression levels of genes implicated in fate conversion between hepatocytes and BECs from the RNA-sequencing data. Intriguingly, we found that YAP target genes, *ccn1* and *ccn2*, were downregulated in GW7647-treated *Tg*(*fabp10a:pt-β-catenin*) livers (Fig. 2A). Using a YAP reporter line, *Tg*(*hCCN2:GFP*)^34^, we also found that YAP activity was highly enhanced in *Tg*(*fabp10a:pt-β-catenin*) livers and that the activity was present in most LPCs, which express Anxa4, an LPC/BEC marker (Fig. 2D). Given a high YAP activity in BECs and ductular reactions^35^, it is possible that the reduced YAP signaling in GW7647-treated *Tg*(*fabp10a:pt-β-catenin*) livers is simply the consequence of enhanced LPC-to-hepatocyte differentiation rather than the consequence of PPARα activation. To exclude this possibility, we examined the expression of *ccn1* and *ccn2* in *Tg*(*fabp10a:pt-β-catenin*) larvae treated with AG1478, an EGFR inhibitor, known to promote LPC-to-hepatocyte differentiation^14^. qRT-PCR analysis showed that AG1478 treatment enhanced the expression of hepatocyte marker genes (*cyp7a1*, *gc, ces2*, *serpina1*) but not YAP target genes (*ccn1* and *ccn2*) (Fig. S2A, B), supporting that PPARα activation suppresses YAP signaling in *Tg*(*fabp10a:pt-β-catenin*) livers. We also validated the reduced YAP signaling identified from RNA-sequencing data with qRT-PCR and Yap1 immunohistochemistry (Fig. 2E, F). Altogether, these findings reveal that PPARα activation suppresses YAP signaling in *Tg*(*fabp10a:pt-β-catenin*) livers.

### Suppression of YAP signaling promotes LPC-to-hepatocyte differentiation, similar to PPARα activation

**Figure 3:**
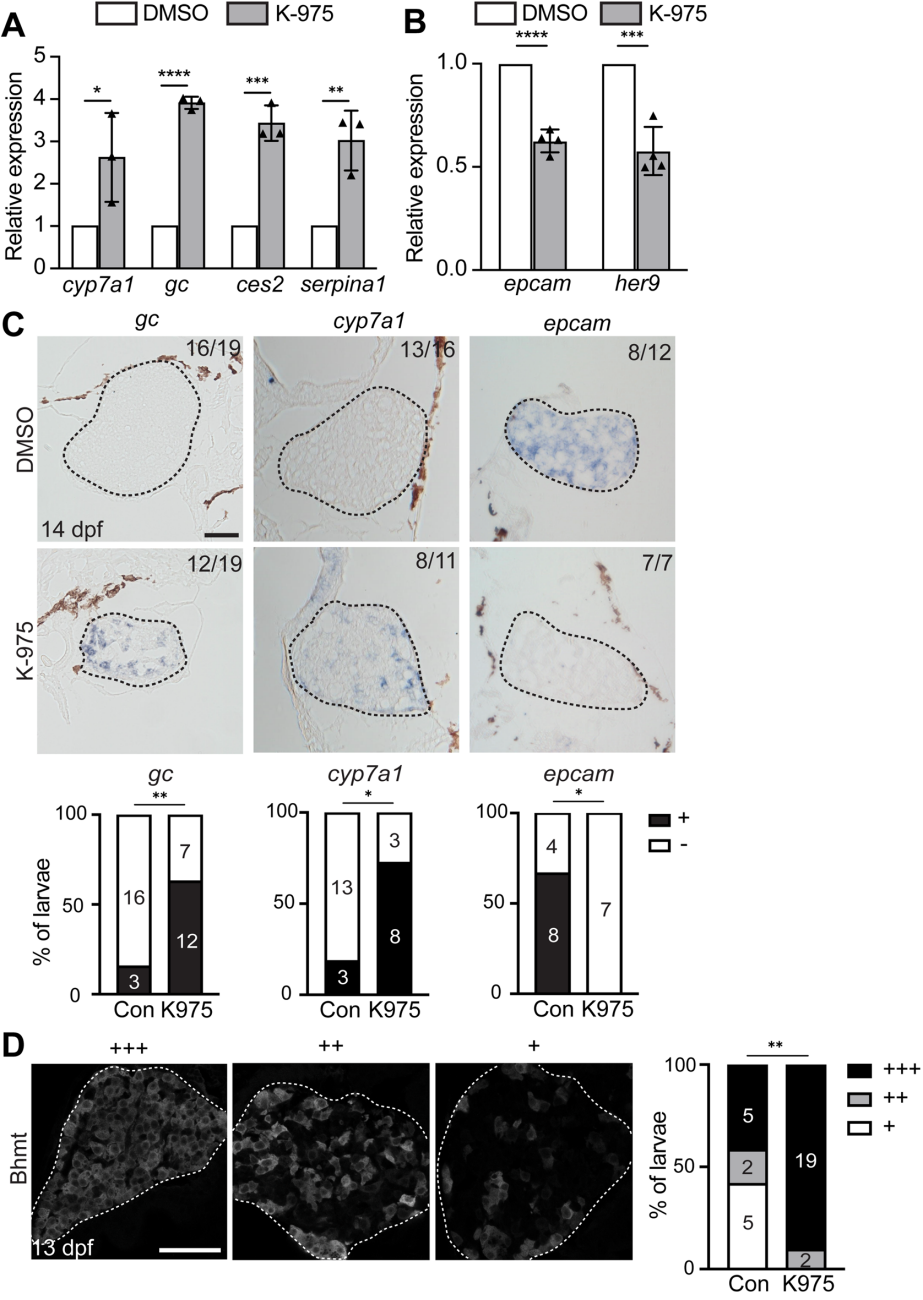
Suppression of YAP signaling promotes LPC-mediated liver regeneration, similar to PPARα activation. (**A**, **B**) qRT-PCR data showing the relative expression levels of hepatocyte (*cyp7a1*, *gc*, *serpina1*, *ces2*) and BEC (*her9*, *epcam*) markers and YAP target genes (*ccn1*, *ccn2*) between DMSO- and K-975-treated *Tg*(*fabp10a:pt-β-catenin*) livers at 14 dpf. (**C**) Section in situ hybridization images showing the expression of hepatocyte (*cyp7a1*, *gc*) and BEC (*epcam*) markers in K-975-treated *Tg*(*fabp10a:pt-β-catenin*) livers at 14 dpf. For quantification, larvae were grouped into +/− based on gene expression levels. Numbers in the upper right corner indicate the proportion of larvae exhibiting the phenotype shown. (**D**) Immunofluorescence images showing Bhmt expression in 13-dpf *Tg*(*fabp10a:pt-β-catenin*) livers treated with K-975. Based on the level of Bhmt expression, larvae were divided into three groups: +++/++/+. Data are represented as mean ± SD (**A**, **B**). *ns* not significant; *P < 0.05, **P < 0.01, ***P < 0.001, ****P < 0.0001; statistical significance was calculated using an unpaired two-tailed *t*-test (**A**, **B**), Fisher’s exact test (**C**), and chi-square test (**D**). Dashed lines outline the livers (**C**, **D**). Scale bars, 50 μm.

Given that YAP signaling positively controls the differentiation of hepatoblasts into BECs^36, 37^ and hepatocyte-to-BEC transdifferentiation^38, 39^, we hypothesized that suppression of YAP signaling might promote LPC-to-hepatocyte differentiation. To test this hypothesis, we suppressed YAP signaling with K-975, the TEAD inhibitor^40^, which suppresses the transcription of YAP target genes by preventing the interaction of TEAD with YAP. In K975-treated *Tg*(*fabp10a:pt-β-catenin*) livers compared with DMSO-treated *Tg*(*fabp10a:pt-β-catenin*) livers, the expression of hepatocyte marker genes (*cyp7a1*, *gc*, *ces2*, *serpina1*) was significantly enhanced and the expression of BEC marker genes (*epcam* and *her9*) was reduced considerably (Fig. 3A, B), as observed in GW7647-treated *Tg*(*fabp10a:pt-β-catenin*) livers. This promotion phenotype was confirmed by in situ hybridization (Fig. 3C) and Bhmt immunofluorescence staining (Fig. 3D). Altogether, these data reveal that suppression of YAP signaling promotes LPC-to-hepatocyte differentiation in *Tg*(*fabp10a:pt-β-catenin*) larvae.

### PPARα activation promotes LPC-to-hepatocyte differentiation by suppressing YAP signaling

**Figure 4:**
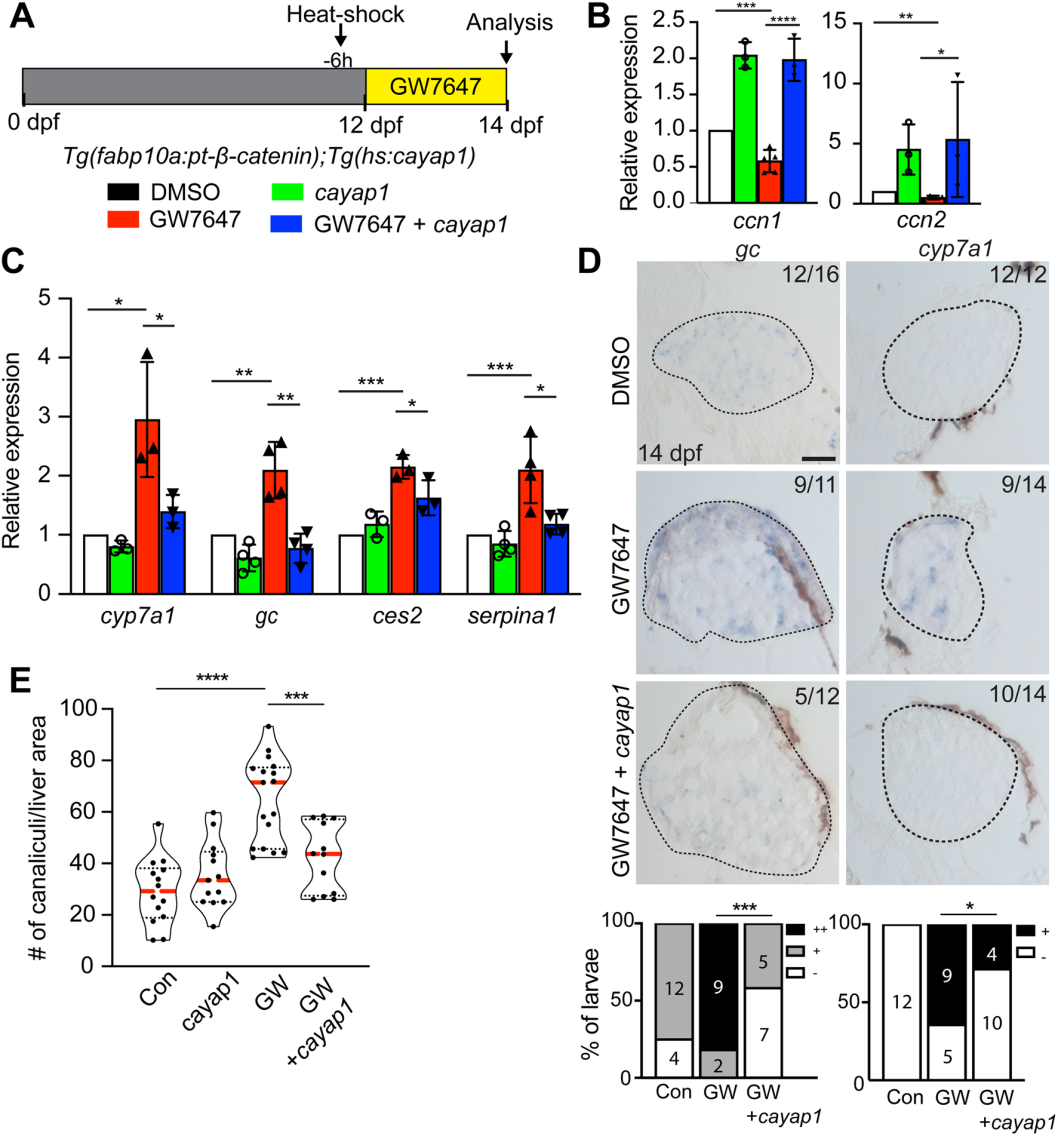
PPARα activation promotes LPC-to-hepatocyte differentiation by suppressing YAP signaling. (**A**) Scheme illustrating an experimental strategy for YAP overactivation. The *Tg*(*hs:cayap1*) line was used to enhance YAP signaling with a heat-shock 6 h before GW7647 treatment. (**B**, **C**) qRT-PCR data showing the relative expression levels of hepatocyte (*cyp7a1*, *gc*, *serpina1*, *ces2*) and BEC (*her9*, *epcam*) markers and YAP target genes (*ccn1*, *ccn2*) among the following four groups of 14-dpf *Tg*(*fabp10a:pt-β-catenin*) livers: (1) DMSO-treated control, (2) *cayap1*^+^, (3) GW7647-treated, and (4) GW7647-treated *cayap1*^+^. Data are represented as mean ± SD. (**D**) Section in situ hybridization images showing the expression of hepatocyte (*cyp7a1*, *gc*) and BEC (*epcam*) markers in 14-dpf *Tg*(*fabp10a:pt-β-catenin*) livers. For quantification, larvae were grouped into ++/+/− based on gene expression levels. Dashed lines outline the livers. Numbers in the upper right corner indicate the proportion of larvae exhibiting the phenotype shown. Scale bars, 50 μm. (**E**) Violin plot graphs showing the quantification of the number of Abcb11^+^ canaliculi per liver area; median and quartiles are indicated by red dashed and black dotted lines, respectively. *ns* not significant; *P < 0.05, **P < 0.01, ***P < 0.001, ****P < 0.0001; statistical significance was calculated using an unpaired two-tailed *t*-test (**B**, **C**, **E**), Fisher’s exact test (**D**; *cyp7a1*), and chi-square test (**D**; *gc*).

To determine if PPARα activation promotes LPC-to-hepatocyte differentiation by suppressing YAP signaling, we performed a rescue experiment by enhancing YAP signaling in *Tg*(*fabp10a:pt-β-catenin*) larvae treated with GW7647. To enhance YAP signaling, the *Tg*(*hs:cayap1*) line^41^, which expresses constitutive-active Yap1 (caYap1) under the heat-shock promoter, was used with a single heat-shock 6 h before GW7647 treatment (Fig. 4A). caYap1 overexpression enhanced the expression of *ccn1* and *ccn2* in both DMSO- and GW7647-treated *Tg*(*fabp10a:pt-β-catenin*) livers (Fig. 4B). Importantly, caYap1 overexpression significantly reduced the effect of PPARα activation on LPC-to-hepatocyte differentiation, as assessed by qRT-PCR, in situ hybridization, and Abcb11b immunostaining (Fig. 4C–E). These rescue data demonstrate that PPARα activation promotes LPC-to-hepatocyte differentiation by suppressing YAP signaling.

### PPARα activation promotes LPC-to-hepatocyte differentiation in the adult hepatocyte ablation model as well

We tested the effect of GW7647 on LPC-to-hepatocyte differentiation in another model of LPC-mediated liver regeneration in which liver regeneration is not caused by β-catenin dysregulation: the hepatocyte ablation model with *Tg*(*fabp10a:CFP-NTR*) fish that express nitroreductase (NTR) specifically in hepatocytes^13^. NTR converts metronidazole (Mtz) into a cytotoxic drug; thus, treating *Tg*(*fabp10a:CFP-NTR*) fish with Mtz ablates hepatocytes that express NTR. In this model, upon massive hepatocyte ablation, BECs first dedifferentiate into LPCs, and the LPCs subsequently differentiate into hepatocytes^13^. *Tg*(*fabp10a:CFP-NTR*) male adults were treated with Mtz for 5 h and subsequently with GW7647 from R1d to R3d for 48 h, and their livers were harvested at R3d for qRT-PCR analysis (Fig. S4A). Although its effects were weaker than those in the *Tg*(*fabp10a:pt-β-catenin*) model, GW7647 treatment increased the expression of two hepatocyte marker genes (*fabp10a*, *gc*) and decreased the expression of the Yap target genes (*ccn1*, *ccn2*) (Fig. S4B), suggesting the enhanced LPC-to-hepatocyte differentiation.

## Discussion

In this study, we present that PPARα activation promotes LPC-to-hepatocyte differentiation by suppressing YAP signaling in LPCs. Although it has been reported that PPARα regulates hepatocyte proliferation-mediated liver regeneration^21, 42, 43^, it is elusive whether PPARα also regulates cell fate conversion-mediated liver regeneration, particularly LPC-to-hepatocyte differentiation. Previous in vitro studies^44, 45^ produced a discrepancy in the role of PPARα in LPC-to-hepatocyte differentiation. Here, using the zebrafish in vivo model, we demonstrate that PPARα activation promotes LPC-to-hepatocyte differentiation. Furthermore, we present the mechanism by which PPARα activation promotes the differentiation: suppressing YAP signaling in LPCs. Given the positive relationship between PPARα and YAP signaling in hepatocytes^21^ and cardiomyocytes^22^, our finding about their relationship in LPCs is rather surprising. However, our rescue experiments with enhanced YAP signaling (Fig. 4) prove that PPARα activation promotes LPC-to-hepatocyte differentiation by suppressing YAP signaling.

It was recently reported that PPARα interacts with YAP signaling in the liver^21^ and heart^22^. Upon PHx, PPARα activation enhances its physical interaction with YAP1 in hepatocytes, thereby facilitating the translocation of YAP1 into the nucleus and subsequent hepatocyte proliferation^21^. During heart development, PPARα enhances Yap1 expression by binding its promoter, thereby driving cardiomyocyte maturation^22^. In both cases, PPARα positively regulates YAP signaling. We also observed in zebrafish that PPARα activation enhanced YAP signaling in the normal liver (Fig. S3). By contrast, we present that PPARα activation negatively regulates YAP signaling in LPCs. These opposite relationships between PPARα and YAP signaling imply that transcriptional regulators could have context-dependent, distinct roles. For example, another nuclear receptor, FXR, promotes hepatocyte proliferation following PHx^46, 47^, whereas it suppresses LPC proliferation in BEC-driven liver regeneration^15^. Different contexts may make PPARα have distinct, or sometimes opposite, effects on YAP signaling.

YAP signaling is one of the crucial signaling pathways in hepatobiliary plasticity^48^. It positively controls hepatocyte-to-BEC conversion and LPC activation, also known as ductular reactions. Hepatocyte-specific overactivation of YAP signaling induced hepatocyte-to-BEC conversion^38, 49–51^, whereas hepatocyte-specific suppression of YAP signaling reduced ductular reactions^35, 52^ and BEC marker expression in hepatocytes^35, 52, 53^. Given this role in hepatocyte-to-BEC conversion, it can be assumed that suppressing YAP signaling may promote BEC-to-hepatocyte conversion or LPC-to-hepatocyte differentiation. Indeed, in vitro experiments showed that suppressing YAP signaling promoted hepatocyte differentiation in LPC lines^54^ and induced hepatocyte-like cells^55^; however, such in vivo evidence has been lacking. Using two zebrafish models of LPC-mediated liver regeneration, *Tg*(*fabp10a:pt-β-catenin*) and *Tg*(*fabp10a:CFP-NTR*), we hereby present in vivo evidence.

In the adult hepatocyte ablation model, we observed rather subtle differences in the expression of hepatocyte markers and Yap1 target genes between DMSO- and GW7647-treated groups compared with those in the *Tg*(*fabp10a:pt-β-catenin*) model. We surmise that these variations could stem from different cellular origins of LPCs between the two models. Specifically, in the *Tg*(*fabp10a:pt-β-catenin*) model, ~ 70% and ~ 30% of LPCs originate from hepatocytes and BECs, respectively^14^, whereas in the adult hepatocyte ablation model, nearly all LPCs originate from BECs^56^. Given that hepatocyte-derived LPCs are distinct from BEC-derived LPCs in gene expression profiles^57^, we speculate that upon GW7647 treatment, hepatocyte-derived LPCs might differentiate into hepatocytes better than BEC-derived LPCs.

In summary, we provide the in vivo evidence that suppression of YAP signaling promotes LPC-to-hepatocyte differentiation. Furthermore, we provide a positive role for PPARα in LPC-to-hepatocyte differentiation. Our study suggests PPARα agonists as potential drugs that cannot only reduce a hepatic lipid level but also promote LPC-mediated liver regeneration.

## Methods

### Zebrafish lines

Zebrafish (*Danio rerio*) were maintained at 28.5 °C on a 14 h light/10 h dark cycle. Embryos and adult fish were raised and maintained under standard laboratory conditions^58^. We used the *ppargc1a*^*sa34243*^ mutant and the following transgenic lines: *Tg*(*fabp10a:pt-β-catenin*)^*s704*^, *Tg*(*fabp10a:CFP-NTR*)^*s931*^, *Tg*(*hs:cayap1*)^*zf622*^, and *Tg*(*hCCN2:GFP*)^*ia48*^. Their full names and references are listed in Table S1. All protocols used within this study were approved by the Institutional Animal Care and Use Committee of the University of Pittsburgh School of Medicine and conform to the National Institutes of Health Guide for the Care and Use of Laboratory Animals. The reporting in this manuscript follows the recommendations in the ARRIVE guidelines^59^. For all experiments, we used embryos from multiple clutches; our breeding strategy is mass breeding to put 3–4 males and 5–6 females in the same breeding tank. We drew conclusions following at least three independent experiments.

### Compound treatments

*Tg*(*fabp10a:pt-β-catenin*) larvae were treated with 0.1% dimethyl sulfoxide (DMSO), 1 µM GW7647 (Cayman Chemical, Ann Arbor, MI), 2 µM K-975 (MedChemExpress, Princeton, NJ), 15 µM Etomoxir (Cayman Chemical, Ann Arbor, MI), 2 µM AG1478 (ApexBio, Houston, TX), or 7 μM CAY10599 (Cayman Chemical, Ann Arbor, MI) for 48 h from 12 or 13 days post-fertilization (dpf). Compounds were refreshed every 24 h. The larvae were fed prior to the drug treatments but fasted during the treatments. They were harvested at 14 or 15 dpf for subsequent analyses.

### The adult hepatocyte ablation model, *Tg*(*fabp10a:CFP-NTR*)

Six month old male *Tg*(*fabp10a:CFP-NTR*) fish were treated with 5 mM Mtz in system water containing 0.5% DMSO for 5 h to ablate hepatocytes. 24 h after Mtz washout (R1d), the fish were treated with 1 mM GW7647 for 48 h and harvested at R3d.

### qRT-PCR

Total RNA was extracted from 30 to 40 dissected livers for each condition using the RNeasy Micro Kit (Qiagen, Hilden, Germany); cDNA was synthesized from the RNA using the SuperScript^®^ III First-Strand Synthesis SuperMix (Thermo Fisher Scientific, Waltham, MA) according to the manufacturer’s protocols. qRT-PCR was performed as previously described^14^ using the QuantStudio 12K Flex machine (Applied Biosystems, Waltham, MA) with the iTaqTM Universal SYBR^®^ Green Supermix (Bio-Rad, Hercules, CA). *eef1a1l1* was used for normalization as previously described^60^. At least three independent experiments were performed. For each replicate with larval livers, the control value was first set to 1, and then the values of the experimental groups were calculated relative to the control value. The primers used for qRT-PCR are listed in Table S3; the Ct values of qRT-PCR data are listed in Table S4.

### RNA-sequencing

Livers were dissected from 30 to 40 DMSO- and GW7647-treated larvae. For adult experiments, the individual liver was dissected without pooling. Total RNA was extracted from the dissected livers using RNeasy Micro kit (Qiagen, Hilden, Germany) and sent to the University of Pittsburgh Genomics Core for library preparation and sequencing. Library preparation was performed using the TruSEQ Stranded mRNA Sample Preparation Kit (Illumina, San Diego, CA) according to the manufacturer’s instructions. Sequencing was performed on a NextSeq 500 (Illumina, San Diego, CA) for 2 × 75-bp paired-end reads. These data have been deposited in NCBI’s Gene Expression Omnibus (GSE226923).

### RNA-sequencing analysis

Raw sequencing data were imported into CLC Genomics Workbench, and reads were mapped to the zebrafish reference genome. Differentially expressed genes (DEGs) used in the pathway analysis were determined between DMSO- and GW7647-treated groups using filters to select genes with Expr Fold Change ≥ |1.5| and Expr False Discovery Rate ≤ 0.05. DEGs were imported into Ingenuity Pathway Analysis to identify signaling pathways and upstream regulators affected by PPARα activation.

### Section in situ hybridization and immunofluorescence staining

Section in situ hybridization was performed as previously described^14^. cDNA from 14- or 15-dpf larvae was used as a template for PCR to amplify genes of interest; PCR products were used for in situ probe synthesis. The primers used for the probe synthesis are listed in Table S2. In the case of *gc*^61^ and *epcam*^62^, their probes were synthesized using plasmids containing the genes. Immunostaining was performed as previously described^14^, using the following antibodies: mouse anti-Bhmt (1:500; a gift from Jinrong Peng at Zhejiang University), mouse anti-Anxa4 (1:300; Abcam, Cambridge, UK), rabbit anti-Abcb11 (1:800; Kamiya Biomedical, Seattle, WA), and Alexa Fluor 488-, 568-, and 647-conjugated secondary antibodies (1:500; Thermo Fisher Scientific, Waltham, MA). For section, larvae were fixed with 4% paraformaldehyde/PBS, embedded in Tissue Freezing Medium (Ted Fella, Redding, CA), and cryo-sectioned to 10-µm thickness.

### Yap1 immunohistochemistry

Zebrafish larvae were fixed with Dietrich's fixative (3.7% formaldehyde/2% glacial acetic acid/30% ethanol) at room temperature for 24 h and processed for paraffin embedding. Paraffin block was prepared as previously described^63^. Samples were cut into 5-µm sections, and the sectioned samples were microwaved for 12 min in pH 6.0 sodium citrate buffer for antigen retrieval. After cooling, samples were placed in 3% H_2_O_2_ for 10 min to quench endogenous peroxide activity. After washing with PBS, slides were blocked with Super Block (ScyTek Laboratories, Logan, UT) for 10 min. Samples were incubated with rabbit anti-Yap1 antibodies (1:00; Cell Signaling, Danvers, MA) and then with biotinylated secondary antibodies (1:500; EMD Millipore, Darmstadt, Germany) for 15 min at room temperature. After washing with PBS, samples proceeded with Vectastain ABC Elite kit (Vector Laboratories, Newark, CA), and the signal was developed with DAB Peroxidase Substrate Kit (Vector Laboratories, Newark, CA). Slides were counterstained with hematoxylin (Thermo Fisher Scientific, Newark, CA) and dehydrated to xylene, and coverslips were applied with Cytoseal XYL (Thermo Fisher Scientific, Newark, CA).

### Heat-shock condition

*Tg*(*hs:cayap1*) larvae were heat-shocked at 12 or 13 dpf 6 h before compound treatments. The larvae were transferred into egg water pre-warmed to 38.5 °C and kept at 38.5 °C for 30 min, as previously described^64^.

### Genotyping of *ppargc1a* mutants

For *ppargc1a* genotyping, genomic DNA was amplified with either a wild-type (5′-CTGTCTTCATGCTCTCCCTC-3′) or mutant (5′-CTGTCTTCATGCTCTCCCTA-3′) allele-specific forward primer and a common reverse primer (5′-TGGGCCCTTCCGAATAGAGC-3′). Both the wild-type and mutant alleles displayed 102-bp PCR products.

### Image acquisition, processing, and statistical analysis

Zeiss LSM700 confocal (Oberkochen, Germany), Leica M205 FA (Wetzlar, Germany), and Nikon Eclipse Ti inverted (Tokyo, Japan) microscopes were used to obtain image data. Confocal images were analyzed using the ImageJ software^65^. All figures, labels, arrows, scale bars, and outlines were assembled or drawn using the Adobe Illustrator software (San Jose, CA). Statistical analyses were performed using the GraphPad Prism software (San Jose, CA). Differences among groups were tested by unpaired Student’s *t*-tests, Fisher’s exact test, or chi-square test. Differences were considered statistically significant when *P* < 0.05 (*P < 0.05, **P < 0.01, ***P < 0.001, ****P < 0.0001).

### Supplementary Information

Supplementary Information.

Supplementary Table S4.

## Data Availability

The RNA-sequencing datasets generated during the current study are available in the Gene Expression Omnibus (GEO) repository, GSE226923.
