# Supplementary material for: PPARα activation promotes liver progenitor cell-mediated liver regeneration by suppressing YAP signaling in zebrafish
Source: Sci Rep. 2023 Oct 25;13:18312. doi: 10.1038/s41598-023-44935-5 (PMC10600117; doi:10.1038/s41598-023-44935-5)
Supplement: Supplementary file 1 — Supplementary Information. [file 41598_2023_44935_MOESM1_ESM.docx]

**Supporting information**

**Supporting figures
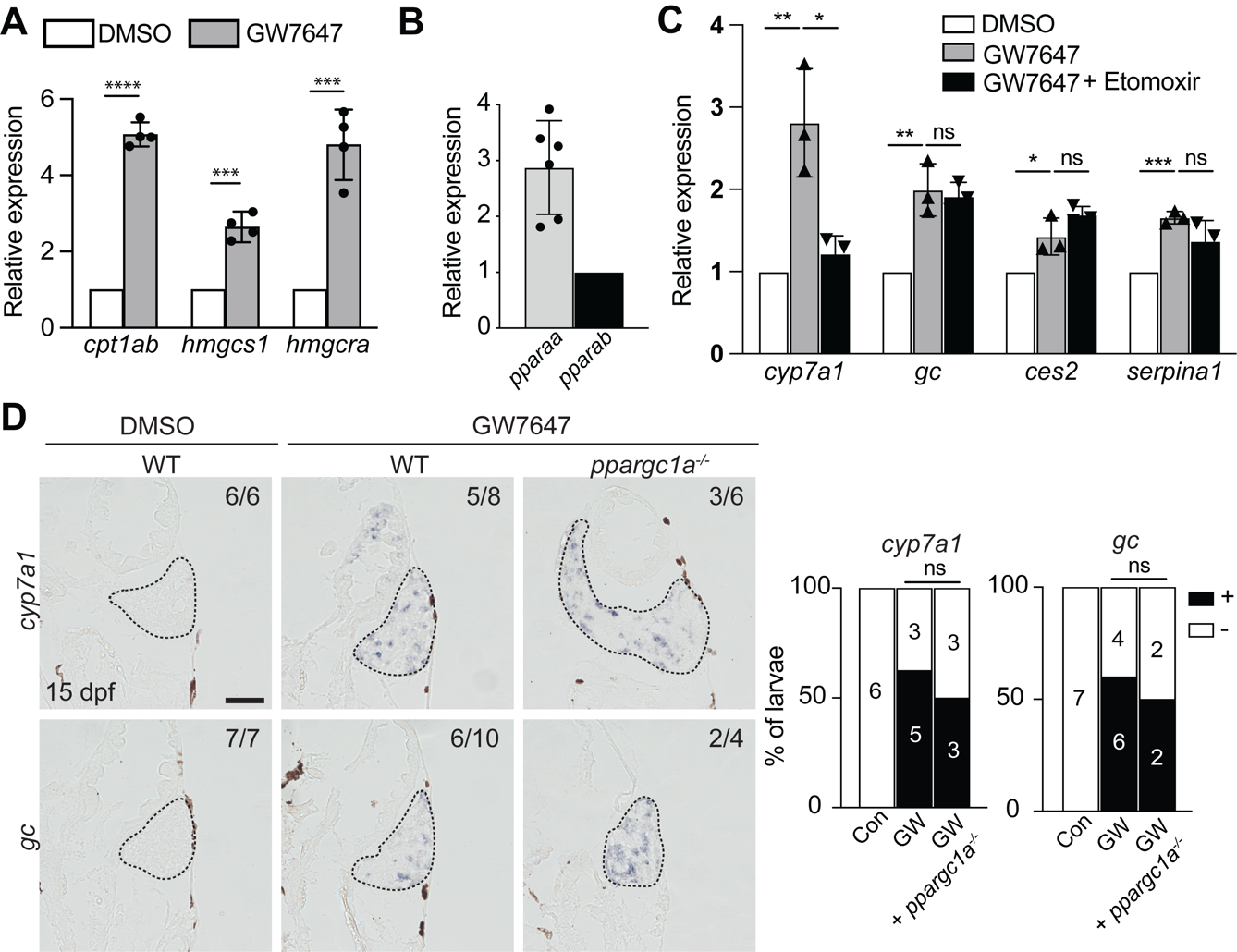
**

**Figure S1.** PPARα activation promotes LPC-to-hepatocyte differentiation not by enhancing FAO. (**A**) qRT-PCR data showing the relative expression levels of PPARα target genes (*cpt1ab*, *hmgcs1*, *hmgcra*) between DMSO- and GW7647-treated *Tg(fabp10a:pt-β-catenin)* livers at 14 dpf. (**B**) qRT-PCR data showing the relative expression levels of *pparaa* and *pparab* in *Tg(fabp10a:pt-β-catenin)* livers at 14 dpf. (**C**) qRT-PCR data showing the relative expression levels of hepatocyte markers (*cyp7a1, gc, ces2, serpina1*) among DMSO-, GW647-, and GW7647/Etomoxir-treated *Tg(fabp10a:pt-β-catenin)* livers at 14 dpf . (**D**) Section *in situ* hybridization images showing *cyp7a1* and *gc* expression in GW7647-treated livers of 15-dpf *Tg(fabp10a:pt-β-catenin)* in wild-type and *ppargc1a* mutant backgrounds. For quantification, larvae were grouped into +/– based on the levels of gene expression. Dashed lines outline the livers. Numbers in the upper right corner indicate the proportion of larvae exhibiting the phenotype shown. Scale bar, 50 μm. ns, not significant; **P*<0.05, ***P*<0.01, ****P*<0.001, *****P*<0.0001; statistical significance was calculated using an unpaired two-tailed t-test (B, C) and Fisher’s exact test (D).


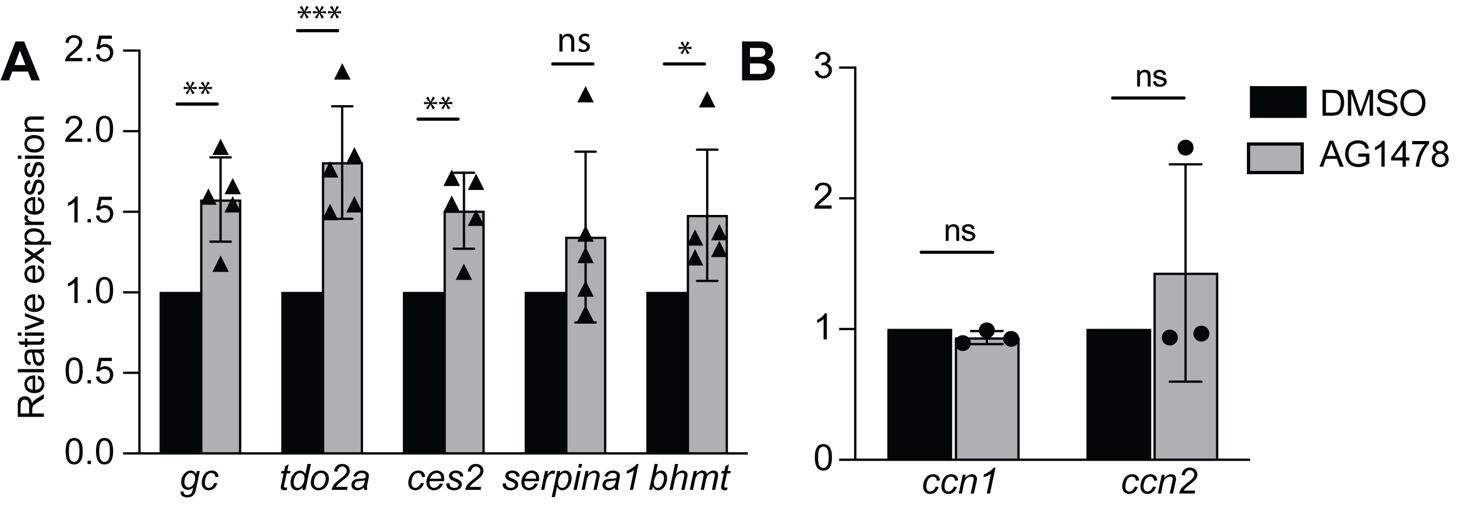


**Figure S2.** EGFR inhibition promotes LPC-to-hepatocyte differentiation without a reduction in YAP signaling. (**A, B**) qRT-PCR data showing the relative expression levels of hepatocyte markers (*gc*, *tdo2a, serpina1, ces2, bhmt*; A) and YAP target genes (*ccn1*, *ccn2*; B) between DMSO- and AG1478-treated *Tg(fabp10a:pt-β-catenin)* livers at 14 dpf. ns, not significant; **P*<0.05, ***P*<0.01, ****P*<0.001; statistical significance was calculated using an unpaired two-tailed t-test.

**
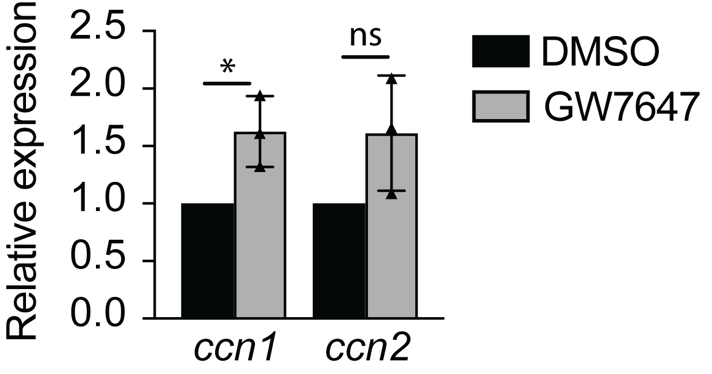
**

**Figure S3.** PPARα activation enhances YAP signaling in the normal liver. qRT-PCR data showing the relative expression levels of YAP target genes (*ccn1*, *ccn2*) between DMSO- and GW7647-treated wild-type livers at 14 dpf. **P*<0.05; statistical significance was calculated using an unpaired two-tailed t-test.


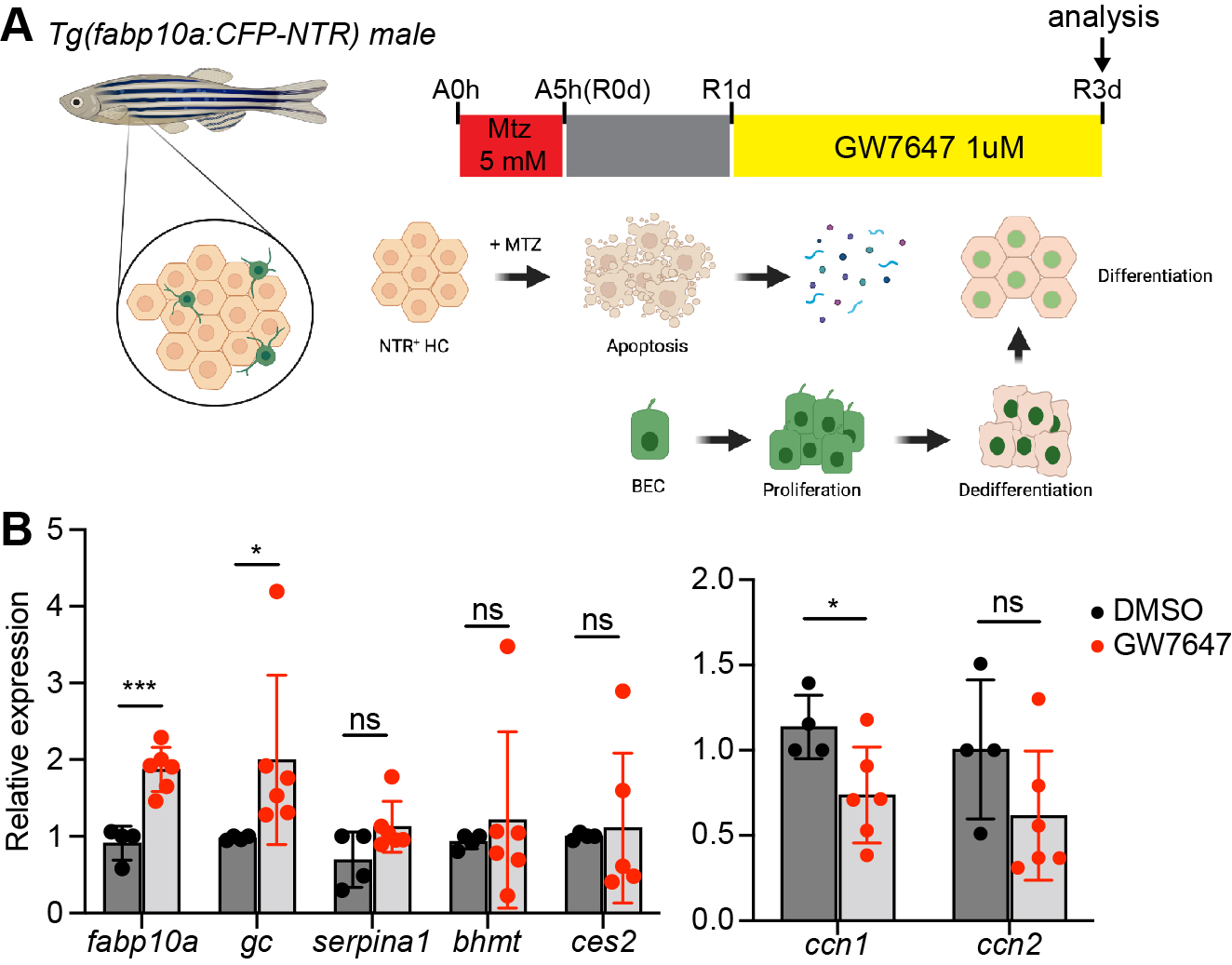


**Figure S4.** PPARα activation promotes LPC-to-hepatocyte differentiation in the adult hepatocyte ablation model. (**A**) Scheme illustrating the experimental strategy and hepatocyte ablation-induced BEC-driven liver regeneration. Six month old male *Tg(fabp10a:CFP-NTR)* fish were treated with 5 mM Mtz for 5 hours to ablate hepatocytes. 24 hours after Mtz washout (R1d), the fish were treated with 1 mM GW7647 for 48 hours and harvested at R3d. (**B**) qRT-PCR data showing the relative expression levels of hepatocyte (*fabp10a*, *gc*, *serpina1*, *bhmt*, *ces2*) and YAP target genes (*ccn1*, *ccn2*) between DMSO- and GW7647-treated adult *Tg(fabp10a:CFP-NTR)* livers at R3d. Data are represented as mean ± SD. ns, not significant; **P*<0.05, ****P*<0.001; statistical significance was calculated using an unpaired two-tailed t-test.

**Supporting Table**

**Table S1. Transgenic and mutant zebrafish lines used in this study**

| Names used in this study | Official names (ZFIN database) | Allele # | Ref |
| --- | --- | --- | --- |
| *Tg(fabp10a:CFP)* | *Tg(fabp10a:CFP-NTR)* | s931 | (1) |
| *Tg(fabp10a:pt-β-catenin)* | *Tg(fabp10a:Xla.Ctnnb1,cryaa:Venus)* | s704 | (2) |
| *Tg(hs:cayap1)* | *Tg(hsp70l:RFP-cayap1)* | zf622 | (3) |
| *Tg(hCCN2:GFP)* | *Tg(Hsa.CTGF:EGFP)* | ia48 | (4) |
| *ppargc1a* | *ppargc1a* | sa34243 | (5) |

**Table S2. Sequences of primers used for *in situ* probe synthesis**

| Gene | Primer | Nucleotide sequence (5' to 3') |
| --- | --- | --- |
| *cyp7a1* | forward | GTTCAGAAAGCTTCACTCTCC |
|  | reverse | TAATACGACTCACTATAGGGGCGTGAACGCCGTAATGAAA |
| *serpina1* | forward | CATGTTGGGTCACAGTCAGG |
|  | reverse | TAATACGACTCACTATAGGGGCCTTTGTAGGGCACCATCAT |
| *pparaa* | forward | GACACTCTCCAGCACGCAG |
|  | reverse | TAATACGACTCACTATAGGGATGCGCTCGATCTGTGG |
| *pparab* | forward | CATGTTGGGTCACAGTCAGG |
|  | reverse | TAATACGACTCACTATAGGGGCCTTTGTAGGGCACCATCAT |

Underlined are T7 primer sequences**.**

**Table S3. Sequences of primers used for qRT-PCR**

| Gene | Primer | Nucleotide sequence (5' to 3') |
| --- | --- | --- |
| *eef1a1l1* | forward | CTGGAGGCCAGCTCAAACAT |
|  | reverse | ATCAAGAAGAGTAGTACCGCTAGCATTAC |
| *cyp7a1* | forward | CCTACCATGCTGTCATCCGTC |
|  | reverse | TCTCATGACCAAATGCCTTCGC |
| *ces2* | forward | GGAATATTAGGATACTTCAGCACAG |
|  | reverse | CTGTAAAGCTGCGATCTGG |
| *gc* | forward | CAAGCATGGGTTTCTTGGG |
|  | reverse | AACCTCCTCTGTCAGTTTCTG |
| *bhmt* | forward | CTGATCGCTGAGTACTTTG |
|  | reverse | CAATGAAGCCCTGGCAGC |
| *serpina1* | forward | CATGTTGGGTCACAGTCAGG |
|  | reverse | CGATTTCAGGCTTGGAGAA |
| *her9* | forward | AATGCCAGCGAGCATAGAAAGTC |
|  | reverse | TGCCCAAGGCTCTCGTTGATTC |
| *epcam* | forward | CTTGTTTGTTGTGGCATTGG |
|  | reverse | TTGACGCACCAGCATACTTC |
| *ccn1* | forward | TCTAGACCTGGAGTGCAACTATGGGG |
|  | reverse | GGAAACTCTCCCCGTTCTGG |
| *ccn2* | forward | GATCAGCTTTAGCAGCTTACAG |
|  | reverse | TCTGAACCAGGCAGTTCTC |
| *yap1* | forward | TGAGATGGAGACAGGTGAC |
|  | reverse | ATGGCGTCTAGGTAATCGG |
| *pparaa* | forward | CGATATTAGATGTCTTAACTCCGGC |
|  | reverse | GATGAGTTCTCGTCCTGACCC |
| *pparab* | forward | ATCATCTGCTGTGGAGATCG |
|  | reverse | GAGAACGTTAACAATGCTCTCCT |
| *cpt1ab* | forward | CCAGCAGATGGAACTCTTTGAC |
|  | reverse | CCATCATCAGCAACAGGTCCA |
| *hmgcs1* | forward | GCCCAAAGATGTGGGAATCATC |
|  | reverse | ACTCCATCATACTCCTCCAGC |
| *hmgcra* | forward | TCAGTCCCAAATTCCCAGAG |
|  | reverse | ATGTGCCCATCACCTTAGAG |
| *fabp10a* | forward | GCAGGTTTACGCTCAGGAGA |
|  | reverse | TCCTGATCATGGTGGTTCCT |
| *tdo2a* | forward | GATGATTGGCAGTAAAGACGG |
|  | reverse | ACCTTGTAGCGATCACTGAC |

**Supporting References**

1. Choi TY, Ninov N, Stainier DY, Shin D. Extensive conversion of hepatic biliary epithelial cells to hepatocytes after near total loss of hepatocytes in zebrafish. Gastroenterology 2014;146:776-788.

2. Evason KJ, Francisco MT, Juric V, Balakrishnan S, Lopez Pazmino Mdel P, Gordan JD, Kakar S, et al. Identification of Chemical Inhibitors of beta-Catenin-Driven Liver Tumorigenesis in Zebrafish. PLoS Genet 2015;11:e1005305.

3. Mateus R, Lourenco R, Fang Y, Brito G, Farinho A, Valerio F, Jacinto A. Control of tissue growth by Yap relies on cell density and F-actin in zebrafish fin regeneration. Development 2015;142:2752-2763.

4. Astone M, Lai JKH, Dupont S, Stainier DYR, Argenton F, Vettori A. Zebrafish mutants and TEAD reporters reveal essential functions for Yap and Taz in posterior cardinal vein development. Sci Rep 2018;8:10189.
